# Comparison of Miniplate and K-wire in the Treatment of Metacarpal and Phalangeal Fractures

**DOI:** 10.7759/cureus.7039

**Published:** 2020-02-19

**Authors:** Zulfiqar Ahmed, Muhammad Imran Haider, M Iqbal Buzdar, Babar Bakht Chugtai, Majid Rashid, Nazar Hussain, Farman Ali

**Affiliations:** 1 Orthopaedics, Bahawal Victoria Hospital, Bahawalpur, PAK; 2 Orthopaedics, Sahiwal Medical College, Sahiwal, PAK; 3 Anaesthesia, DHQ Khanewal, Khanewale, PAK

**Keywords:** k-wire, miniplate, metacarpal, phalangeal fracture, range of motion.

## Abstract

Introduction

Metacarpal and phalangeal fractures are common upper limb fractures due to direct blows, axial loading, and torsional loading injuries. The universal goal in treating all fractures for the patient to achieve normal motion, but the ideal technique for stabilization is still debated. For internal fixation, Kirschner wires (K-wires) or miniplates can be used, and each carries certain advantages. No previous study has compared K-wire use to miniplate use in treating metacarpal and phalangeal fractures. Therefore, we conducted this randomized control trial to evaluate the outcomes of K-wire and miniplate use in treating metacarpal and phalangeal fractures.

Materials and methods

This randomized controlled trial was conducted in the Department of Orthopaedic Surgery, Bahawal Victoria Hospital, from February 2017 to February 2018. Seventy-five patients were included in this study and randomly assigned into two groups. One group was treated with K-wire fixation, and the other group was treated with miniplate fixation. We assessed total active motion (TAM), range of motion (ROM), duration of injury, and complication rate. Data were analyzed using IBM SPSS Statistics for Windows, Version 23.0 (Armonk, NY: IBM Corp). P values ≤ 0.05 were considered significant.

Results

Mean surgical time, pain scale, and time of union of K-wire treated patients was 38.63±3.64 minutes, 4.17±1.11, and 12.95±3.38 weeks, respectively. The success of the union was noted in 38 K-wire patients (95%). Total active ROM was greater in miniplate fixation patients compared with K-wire treated patients, but this difference was statistically significant. Similarly, TAM was also greater in the miniplate fixation patients compared to the K-wire treated patients, but this difference was also not statistically significant.

Conclusion

Both K-wire fixation and miniplate fixation are equally effective in terms of TAM, ROM, and complications when used to treat metacarpal and phalangeal fractures.

## Introduction

Metacarpal and phalangeal fractures are common fractures of the upper limb associated with permanent loss of upper extremity dexterity [1]. The bones of metacarpal and phalanges are tubular with collective transverse arches and intrinsic longitudinal arches [2]. These bones are attached at the proximal and distal ends with ligamentous attachments and are concave on the palmer surface. Injury from direct blows, axial loading, and torsional loading are the main causes of metacarpal shaft fractures [3].

Displacement and any neurological and vascular injuries must be treated with early mobilization through open reduction and internal fixation to achieve maximum normal function and anatomy [4]. Early mobilization reduces joint stiffness, edema, and adherence to free normal gliding structures [5]. In every type of fracture, the ultimate goal is to achieve normal motion, but the technique of choice for stabilizing the fracture remains debated [6].

The ideal technique for stabilization and fixation would have a minimum cost, maximum bony union, and allow for proper rotation, length, alignment, and recovery of normal hand functioning without fear of displacement [7]. In internal fixation, Kirschner wires (K-wires) or miniplate can be used. K-wire use has some advantages over the miniplate approach such as advanced material, minimum dissection, technical ease, and availability of the material [8]. The miniplate, however, has modifications specially designed for hand surgery. Through the use of these specially designed plates, we can achieve extra rigidity and length maintenance [9,10].

No previous studies are available that compare K-wire outcomes with miniplate outcomes in treating metacarpal and phalangeal fractures. We conducted this randomized control trial to evaluate the outcomes of K-wire use with miniplate use in treating metacarpal and phalangeal fractures.

## Materials and methods

This randomized controlled trial was conducted in the Department of Orthopedic Surgery of Bahawal Victoria Hospital, Bahawalpur, from February 2017 to February 2018. The study received ethical approval from our institutional ethics board, and informed consent was obtained from all patients studied. The study included all displaced fractures of the metacarpal and phalangeal bones with malrotation, irreducible, subcapital open fractures, intra-articular fractures, multiple traumas of the hand, and segmental bone loss presenting to the emergency ward. Patients with osteoporosis and firm cortical acquisition were excluded from the study. Non-probability consecutive sampling was used. Patients were divided into two equal groups randomly. One group was treated with K-wire fixation, and the other group was treated with miniplate fixation.

Patients were treated under digital block and mild sedation to flex and extend the fingers for assessment of digit movement. A dorsal skin incision exposed the fracture site, and the periosteum was incised longitudinally enough to cleanse and place the desired fixation. The fixation pin was drilled in retrograde from the fracture margin along the dorsoradial aspect of the metacarpal, then back down the shaft to reduce the fracture. The pin was inserted, and rotational alignment was maintained. The patient was asked to flex and extend the digits. If alignment was not proper, the pin was removed, and another pin was inserted. After surgery, a bulky dressing was done for five days, and the patient was asked to engage in active mobilization. Heavy lifting and gripping were not allowed for six weeks following the surgical procedure.

For metacarpal fractures, the site was exposed with a direct incision on the border of the radius and the first two metacarpals, and on the border of the ulna and the fifth metacarpal. A longitudinal incision was made between the third and fourth metacarpals to expose the fracture site. A mid-lateral incision exposed the phalangeal fracture. A lateral band was incised for optimum exposure of the proximal phalanx. the periosteum was incised and exposed by elevating the fracture to avoid violation of the gliding space, the periosteum, and the extensor tendon. The drill hole was made at the correct position in a single attempt. When fixation was secured, the original position of the periosteal layer was returned and sutured if needed. Soft dressing was applied after wound closure, and one day following the procedure, an active range of motion (ROM) exercise was started under the supervision of the surgeon.

The follow-up period was eight weeks. The surgical site was evaluated weekly, and x-rays were taken to observe the loss of reduction and monitor bone healing. Active ROM was measured, and patients were discharged once ROM peaked and the fracture had healed significantly. Data were analyzed using IBM SPSS Statistics for Windows, Version 23.0 (Armonk, NY: IBM Corp). We evaluated pain via the visual analogue scale, surgical time, success of the union, and complications. The mean and standard deviation was calculated for numerical data and frequency (percentages) were calculated for qualitative data. P-values of less than 0.05 was taken as statistically significant.

## Results

Seventy-five patients (42 men, 33 women) were enrolled in this study. Forty patients (21 men, 19 women) were treated with K-wire fixation, and 35 patients (21 men, 14 women) were treated with miniplate fixation. The mean age and time injury of K-wire treated patients was 25.20±1.78 years and 4.38±1.44 hours, respectively. There were 12 open fractures (30%) and 17 intraarticular fractures (42.5%) in the K-wire group. For the miniplate group, the mean age and time injury was 25.51±2.18 years and 4.88±1.23 hours, respectively. There were nine open fractures (25.7%) and 16 intraarticular fractures (45.7%) in the miniplate group. The distribution of fracture categories was not statistically significant and is shown in Table 1.

**Table 1:** Demographic data

Variable	K-wire (n=40)	Miniplate (n=35)	P-value
Gender			
Male	21 (52.5%)	21 (60%)	0.514
Female	19 (47.5%)	14 (40%)	
Age (years)	25.20±1.78	25.51±2.18	0.496
Time injury (hours)	4.38±1.44	4.88±1.23	0.106
Open fracture	12 (30%)	9 (25.7%)	0.680
Intraarticular	17 (42.5%)	16 (45.7%)	0.780
Fracture category			
Metacarpal	16 (40%)	15 (42.9%)	0.789
Phalanx	25 (50%)	18 (51.4%)	
Thumb	4 (10%)	2 (5.7%)	

The mean surgical time, pain scale, and time of union of K-wire treated patients was 38.63±3.64 minutes, 4.17±1.11, and 12.95±3.38 weeks, respectively. Thirty-eight fractures had a successful union (95%) shown in Table 2. The mean surgical time, pain scale, and time of union of miniplate fixation patients were 52.34±3.45 minutes, 5.25±1.57, and 11.80±2.38 weeks, respectively. Thirty-four fractures had a successful union (97.1%). Total active ROM was greater in miniplate fixation patients compared with K-wire patients, but this difference was not significant (Table 2). Similarly, total active motion (TAM) was also greater in miniplate fixation patients compared with K-wire treated patients, but the difference in TAM was not statistically significant. The distribution of different complications of both groups is presented in Table 3.

**Table 2:** Study outcome variables MP:metacarpophalangeal joint; PIP: proximal interphalangeal joint; DIP: distal interphalangeal joint.

Variable	K-wire (n=40)	Miniplate (n=35)	P-value
Outcomes			
Surgical time (minutes)	38.63±3.64	52.34±3.45	0.000
Pain scale	4.17±1.11	5.25±1.57	0.102
Time of union (weeks)	12.95±3.38	11.80±2.38	0.485
Success of union	38 (95%)	34 (97.1%)	0.637
Average ROM (degree)			
MP			
Normal	--	--	--
Metacarpal	76.95±2.57	85.71±1.94	0.071
Phalanx	79.32±2.51	78.28±3.06	0.210
Thumb	76.57±1.79	82.62±2.93	0.354
PIP			
Normal	--	110.82±3.39	--
Metacarpal	101.05±4.95	105.48±3.11	0.101
Phalanx	96.90±5.65	97.62±1.78	0.421
Thumb	95.80±4.05	97.51±2.02	0.085
DIP			
Normal	--	64.25±1.94	--
Metacarpal	62.25±1.99	64.31±2.60	0.324
Phalanx	59.95±2.84	62.77±3.78	0.357
Thumb	--	--	--
Total active motion			
Normal	--	260.20±4.49	--
Metacarpal	246.63±3.22	254.37±3.44	0.142
Phalanx	216.43±4.18	242.34±6.20	0.247
Thumb	210.35±5.26	234.34±6.49	0.094

**Table 3:** Complications

Complications	K-wire (n=40)	Miniplate (n=35)	p-value
Infection	4 (10%)	1 (2.9%)	0.825
Implant loosening	2 (5%)	1 (2.9%)	
Loss of reduction	3 (7.5%)	2 (5.7%)	
Stiffness	4 (10%)	3 (8.6%)	
Malunion	2 (5%)	2 (5.7%)	
Total	15 (37.5%)	9 (25.8%)	

## Discussion

James et al. reported that 77% of fingers lost function when treated with closed reduction of an unstable phalangeal fracture [11]. These results were unsatisfactory due to the loss of active ROM at the phalangeal joint with K-wire fixation; only 8% of patients regained full phalangeal functions. James et al.’s results are comparable to our findings. Green et al. reported 69% of patients treated with closed reduction and percutaneous pin fixation of proximal phalangeal fractures had satisfactory results. They concluded that this technique was only useful for oblique fractures of the proximal phalanx [12].

One study reported 32 patients with hand fractures due to injury from low-velocity bullets were treated by cleansing the wound, debridement, splinting, and use of antibiotics [13]. No infection occurred, but unstable fractures yielded poor ROM at their associated joints-which aligns with our findings. Barton et al. reported 57% satisfactory results in patients with comminuted fractures, which also aligns with our results [13].

Huffaker et al. conducted a study on factors influencing reductions in ROM [14]. They reported satisfactory results in 67% of patients without considering any specific method of treatment. They reported a 20% decreased ROM in unfractured phalangeal bones of similar hands. Injuries that involve extensor and flexor tendons affected the end results. Strickland et al. reported that immobilization for longer than two weeks might decrease TAM [15]. About 25% of phalangeal shaft fractures treated with open reduction and fixation with K-wires yielded good TAM of 142°.

Lister et al. reported that phalangeal shaft fractures treated with K-wire fixation and immobilization of three weeks yielded improved results of nearly 157° TAM. Lister et al. achieved a TAM of 199° and recommended K-wire fixation as the treatment of choice [16]. Belsky et al. concluded that closed reduction with internal fixation is better than open reduction-they observed excellent results in 69% of cases, 29% had good results, and 10% had fair to poor results. Belsky et al.’s findings are important and add to the debate on the proper management of phalangeal fractures [17].

Miniplate is a biomechanically effective tool for surgeons when a fracture is located at the phalangeal shaft. The miniplate is more flexible than K-wire fixation, which can slide through the bony union. In a study by Fyfe et al., fixation was found to be weaker when K-wire was used. Fyfe et al. found that fixation with miniplate yielded much better results [18]. Massengill et al. reported that miniplate fixation provides equal stabilization and results in better ROM and outcomes of TAM. They found that achieving normal phalangeal function was more consistent with miniplate use [19].

Miniplate provides significant stabilization, so mobilization and exercises for active ROM can start sooner. Soft dressing should be applied to allow for the early commencement of activities. Wutphiriya-angkul et al. reported that both K-wire and miniplate techniques are equally effective in treating metacarpal and phalangeal fractures [13]. Both techniques required minimum time for the operation, and findings from that study are identical to ours in that both techniques are time-saving and equally effective when used in metacarpal and phalangeal fractures.

## Conclusions

Both techniques K-wire fixation and miniplate fixation are equally effective in terms of TAM, ROM, and complications when used to treat metacarpal and phalangeal fractures.
